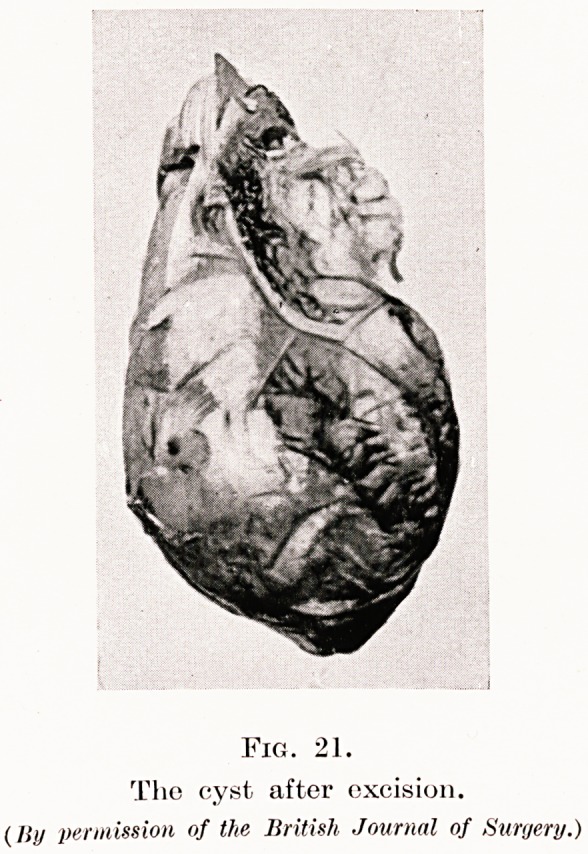# Thoracic Surgery
*A Paper read before the Bristol Medico-Chirurgical Society on Wednesday, 12th January, 1938.


**Published:** 1938

**Authors:** A. L. d'Abreu

**Affiliations:** Senior Assistant in the Surgical Unit, Welsh National School of Medicine, Cardiff; Assistant Thoracic Surgeon, Welsh National Memorial Association, Cardiff


### THORACIC SURGERY.*

BY

A. L. d'Abreu, Ch.M., F.R.C.S.,

*Senior Assistant in the Surgical Unit, Welsh National School of Medicine, Cardiff;*

*Assistant Thoracic Surgeon, Welsh National Memorial Association, Cardiff.*

---

The scope of modern thoracic surgery is so great that I must limit myself to a brief description of the principles underlying the treatment of acute and chronic empyema, pulmonary tuberculosis, bronchiectasis and lung abscess, and of some intra-thoracic tumours and cysts. The scope of thoracic surgery depends largely on the general practitioner, the general physician, and the tuberculosis officers; with regard to the last group, it must not be thought that their field includes only tuberculosis. Indeed, most of the non-tuberculous patients about whom I shall talk were referred to me by officers of the Welsh National Memorial Association. It is of increasing importance that the physician should be surgically-minded, just as the thoracic surgeon must have a sound knowledge of the pathology and physical signs of chest disease, and be able to interpret accurately the radiograms: if he is merely a technical expert dealing with the mechanical problems of the chest his patients will be in constant jeopardy.

*A Paper read before the Bristol Medico-Chirurgical Society on Wednesday, 12th January, 1938.

In addition to the wise selection of patients by his colleagues and himself the thoracic surgeon is dependent largely on the goodwill of hospital and tuberculosis authorities. This slide shows the radiograph of a patient with a chronic empyema of ten years' duration extending almost from the dome of the pleura to the diaphragm (Fig. 8). Such tragic patients are chronic in every sense of the word: they need prolonged drainage, perhaps for six months, and this often has to be followed by collapse operations. This girl had to undergo a very complete thoracoplasty to cure her. Her stay in hospital was nine months. If hospital authorities are to encourage thoracic surgery they must view such problems with full sympathy.

With regard to the tuberculous patient requiring surgical treatment, the difficulties are great in many areas. South Wales is particularly fortunate in this respect, for the Memorial have in Sully Hospital a completely modern chest hospital of three hundred beds, and it is fully equipped to deal with any major thoracic operation. Here the patients can be treated along sanatorium lines before being operated upon, and this is infinitely preferable to the practice in many areas of sending patients from the sanatorium to a general hospital for their operation, for these subjects become peculiarly sensitive to their surroundings, and psychologically are extraordinarily dependent upon the personalities of their medical and nursing attendants. The undoubted value of bed-rest and the sanatorium régime is universally proved: there is unfortunately a tendency to regard the provision of such facilities as sufficient for the care of the consumptive, and in many districts the advantages of supplementing this type of treatment by suitable surgery are not realized.

### ACUTE EMPYEMA.

This condition should not be treated as an acute surgical emergency: nothing can be more disturbing to an ill patient than needling and drainage of the pleural cavity immediately on arrival in hospital. The three main problems are diagnosis, when and where to drain, and by what method (*i.e.* closed or open drainage), and when to remove the drainage tube.

*Diagnosis.*—I need not detain you with a description of the physical signs. It is well to remember, however, that there has been a gradual decrease in incidence of typical lobar pneumonia with its classical crisis: instead, we see a more diffuse type of lobular pneumonia in which the onset of purulent effusion is more insidious and more liable to escape detection. No longer is the first sign of an empyema made by observing on the temperature chart a gradual return of pyrexia after the dramatic crisis. Broncho-pneumonia almost from the start may be complicated by an effusion (syn- or para-pneumonic empyema). In influenzal epidemics patients may be up and about their daily duties with the chest full of fluid. It is foolish to refuse help from the radiologist, however definite the physical signs: I have found the use of X-rays to be of inestimable value both in diagnosing apical, interlobar and mediastinal empyema and in enabling the optimum site for drainage to be estimated.

*When to drain an empyema.*—The addition of an open pneumothorax after rib resection may prove fatal to a patient suffering from pneumonia and effusion, since vital capacity may be reduced to a dangerously low level because of mediastinal flutter. When an empyema accompanies pneumonia the effusion is often due to the streptococcus, and is thin and grey (pyothorax rather than a localized pleural abscess). In such types the mediastinum is not rigid or adherent. Drainage by rib resection and pleurotomy should only be used when there is frank pus present; such a condition is present in classical pneumococcal empyema (meta-pneumonic empyema) or when a thin streptococcal effusion becomes thicker after repeated aspiration.

*Aspiration of purulent effusions.*—To relieve respiratory embarrassment and to remove toxic fluid is an obvious duty even if rib resection is contra-indicated. It is important that the aspiration should be done without allowing air to pass into the chest and to cause further pulmonary collapse. A Potain's or Dieulafoy's aspirator should be used. If these are not available the following method is simple and safe. To a 20 c.c. Record syringe a piece of rubber tubing is attached and connected with a large bore exploring needle (Fig. 1). When the syringe is full of pus an artery forceps is placed on the rubber tube: the syringe is removed and emptied. The artery clip is retained in position until the syringe has been re-attached to the tubing; the manœuvre is repeated as often as necessary.

In nervous or young patients the repeated pricks in the skin become irksome and unbearable: this suffering can be avoided by making a small incision under local anæsthesia over the intercostal space and packing the wound open with flavine gauze: this is removed each time aspiration is done. (Tudor Edwards.) It is remarkable that by this method the parietal pleura becomes insensitive and injections of novocain before each aspiration are unnecessary.

*Intercostal drainage.*—Occasionally this is necessary before rib resection and indeed in a few instances it may replace it. In ill patients the tube is inserted in the ward and I have found the following method simple and effective. A Hamilton Bailey suprapubic trocar, a Malecot's catheter with reinforced tip, and a large bottle with a rubber bung with two openings are required: the bottle contains a little antiseptic fluid into which a long glass tube runs: a smaller glass tube occupies the other hole in the cork. The whole apparatus is assembled as shown in Fig. 4. Under local anæsthesia a small incision is made between the two ribs and the trocar armed with the catheter is inserted through anæsthetized pleura: the trocar is withdrawn and the catheter is left fitting snugly in the chest: air-tight drainage is thereby established.

*Drainage by rib resection.*—When the indications are present this should not be delayed, as early evacuation of the pus encourages lung expansion, which is the essential factor in obliterating the empyema cavity. There is little doubt that for most empyemata closed drainage into a water-sealed bottle by a tube of the Tudor Edwards type (Figs. 2 and 5) is preferable to open drainage by a tube into dressings, since it is cleaner, makes frequent dressings unnecessary, and especially because it re-establishes a negative pressure in the pleural cavity and this encourages lung expansion. Irrigation by means of the small side tube is also possible from the start, and this clears away any fibrin and thick pus. In routine cases irrigation with eusol solution along the small tube is done from the first day.

The site of drainage is important: usually this should be just above the base of the empyema and in the posterior axillary line: if too low the tube becomes blocked by the diaphragm (which usually comes up a little) and if too far back (a common mistake) the tube becomes blocked by the patient lying against it. Apical, interlobar and mediastinal empyemata are drained at appropriate sites (Fig. 6). In the after-treatment respiratory exercises are practised under the control of a masseuse to encourage active respiratory expansion of the lung. Blowing exercises (so often prescribed) are of little value, as the movement to encourage is inspiratory rather than expiratory.

*When to remove the tube.*—The commonest cause of chronic empyema is too early removal of the drainage tube. Frequently the closed method is abandoned after about a fortnight and an open tube used in its place. This must not be dispensed with until the surgeon is sure there is no longer a cavity present. To remove the tube just because pus has ceased to drain is incorrect. Although there are several methods of deciding when the lung has completely expanded, radiography after the injection of lipiodol into the cavity is by far the most reliable, as this gives absolute evidence of the presence or absence of an empyema cavity.

### PLATE I.

Fig. 1.

Method of aspirating a purulent effusion when a Potain's or Dieulafoy's apparatus is not available.

Fig. 2.

Tube fixed in position.

The tube can be fitted snugly against the skin of the chest by the use of elastic adhesive strap passed over the outer flange of the tube.

Fig. 3.

Diagrammatic representation of extra-pleural artificial pneumothorax employed for treatment of a cavity.

### PLATE II.

Fig. 4.
Apparatus assembled for intercostal drainage.

The catheter and trocar are made by the Genito-Urinary Manufacturing Co., Devonshire Place, London.

Fig. 5.
Tudor Edwards's type of drainage tube.

Fig. 6.
Site at which an apical empyema was drained.

Fig. 7.
A patient with an empyema sinus that had been present for 10 years: the site of drainage was too far forward.

### CHRONIC EMPYEMA.

Three common types may be mentioned: patients who have never been diagnosed and drained (Fig. 9), those who have been inadequately drained because the tube has been placed in a faulty position (Fig. 7), or more commonly has been removed before the cavity has become obliterated, and those due to tuberculous disease. A bronchial fistula and a foreign body such as a drainage tube that has escaped unnoticed into the cavity are also causes, while in the last year I have seen three patients suffering from actinomycosis with persistent empyema fistulæ. The history of these patients is often typical, a common cold being followed by a return of discharge through a partially healed wound or by the sudden appearance of foul sputum when there is a bronchial fistula present.

*The investigation of chronic empyema.*—The pus must always be carefully examined for the tubercle bacillus and lipiodol should be run into the sinus and the chest X-rayed. Figs. 10 and 11 are radiograms of a chronic empyema before and after lipiodol injections. Sometimes the plain X-ray alone is sufficient, as in Fig. 8, which represents the chest of a child of twelve who for eight years had suffered from an empyema which had discharged either as the result of spontaneous breaking down of the scar or as the result of operation. The picture is that seen after prolonged drainage and decortication of the lung. The lung would not come out and a ten-rib thoracoplasty was required to close the enormous cavity.

*Treatment of chronic empyema.*—This is a long, tedious matter, and the patient may require to be in

E

hospital for many months. The first requisite is adequate redrainage by the closed method, preferably associated with suction drainage: in many patients it is surprising how well the lung will expand after years of collapse, especially when deep breathing exercises are employed under the eye of a competent masseuse. If the lung will not expand after several months' treatment, then further measures such as thoracoplasty, often combined with the turning in of a large hinged flap containing parietal pleura and muscle (Roberts's operation), are necessary. These operations are formidable, and very large portions of ribs back to the transverse processes of the vertebræ must be excised. If the cavity extends as far as the third rib posteriorly the first rib must be excised. With smaller cavities a local thoracoplasty suffices.

### PULMONARY TUBERCULOSIS.

Bed-rest and a regulated régime are the sheet-anchors of treatment. As an adjuvant to general sanatorium treatment, surgery is often a life-saving help to patients with productive tuberculous disease who are showing a tendency by their own efforts at fibrosis to heal their disease. Although surgical measures, such as phrenicectomy and multiple intercostal neurectomy, are sometimes of help in certain exudative lesions, I shall confine my remarks mainly to the treatment of fibro-cavernous disease.

It is quite certain that some cavities will and do heal under bed-rest treatment alone. If after some months, however, the cavity is stationary or enlarging, and the lesion is unilateral or with moderate disease on the other side that can be controlled and the patient is of suitable age (fifteen—forty-five) surgical help is often required. Although many patients live in fair comfort with open cavities, these must be regarded as an ever-present danger, on account of the risk of spreading to other parts, secondary infection and hæmoptysis. The surgery of pulmonary tuberculosis aims at producing relaxation of the diseased lung: there is no doubt that usually the best way to achieve this is by an effective artificial pneumothorax which collapses the lung and yet leaves a negative pressure in the pleural cavity.

*Surgical assistance to a pneumothorax.*—If the pneumothorax does not control the cavity or lesion by providing a selective collapse it is often quite useless. Such collapses may be prevented by adhesions which can be divided by means of the thoracoscope under local anæsthesia. The operation may be a difficult one and requires great patience and judgment. Complications, of which the commonest is an effusion, are now quite uncommon with a wise selection of cases. Where the adhesions are too dense for division by intrapleural pneumolysis, a phrenic nerve interruption may help especially for so-called "hanging" cavities held up towards the apex by adhesions and with the partly collapsed lung bound down to the diaphragm below. I believe the operation is not used sufficiently often in this type of patient. Where a pneumothorax is desirable but cannot be obtained because of dense adhesions, the operation of extra-pleural artificial pneumothorax may be employed with great advantage.

*Phrenic nerve operations.*—The nerve may be avulsed (Felix operation), the tearing out of the nerve being done to ensure division of the frequently present accessory nerve which runs to it from the nerve to the subclavius: or it may be divided and the accessory nerve also cut (radical phrenicectomy) or finally the "crush" operation is often employed. This last measure is of great value when the effects of hemi-diaphragmatic paralysis are speculative. For example, we know that a phrenic paralysis may close a soft-walled apical cavity, but far more commonly it does not do so. Should it fail, a local upper thoracoplasty or an extra-pleural artificial pneumothorax may be successful. If such local operations are employed it is obviously a great advantage to have the diaphragm moving so that the normal lower lobe may be retained for its respiratory value, and because there is evidence that an upper thoracoplasty performed on a patient with a paralysed diaphragm is often followed by massive collapse of the lower lobe. The advantages of phrenic crush then are that it is not so irremediable (function being often regained in six months), and if it fails to have its desired effect the return of function is allowed: if, however, the crush is followed by satisfactory results the effects are made permanent by proceeding to an avulsion or radical phrenicectomy.

It is impossible to describe in detail the indications for phrenic nerve operations, but in the past too much has been expected of this operation. Its uses are:—

1. To provide rest when the lung is in the state of early productive disease with young fibrous tissue, the contraction of which will often be greatly helped by diaphragmatic relaxation.

2. To supplement an artificial pneumothorax and especially at the end of artificial pneumothorax treatment when the lung is being allowed to creep out; for

### PLATE III.

FIG. 8.

A chronic empyema of many years' duration. After adequate redrainage for many months the lung would not expand and thoracoplasty was necessary.

FIG. 9.

A large chronic empyema. Lipiodol has been injected into the trachea. At operation the walls of the empyema were found to be calcified.

FIG. 10.

FIG. 11.

A plain X-ray and one taken after lipiodol has been run into the sinus of the patient shown in Fig. 7. In Fig. 11 note that although posterior segments of rib have been removed and the empyema has been redrained, there is still a large cavity left. This was later closed by Roberts's method.

### PLATE IV.

FIG. 12.

Ten rib thoracoplasty for fibro-cavernous tuberculosis.

FIG. 13.

Radiograph showing a collapsed upper lobe due to an adenoma of its bronchus.

FIG. 14.

FIG. 15.

Congenital cystic disease of the right upper lobe before and after lipiodol bronchography. (Sully Hospital.)

the dimensions of the pleural cavity into which the previously diseased lung must expand can thus be decreased.

3. As symptomatic treatment, *e.g.* for relief of a non-productive cough due to diaphragmatic adhesions, of pain after pleurisy, or of tachycardia due to pericardial adhesions.

4. For the collapse of cavities by relaxation. There is no doubt at all that the operation may be followed by closure of quite large cavities. I do not want you to think, however, that I am too sanguine about the prospects of cavity closure by phrenicectomy: the results are good in a few cases, but many more have cavities uninfluenced than have them closed.

5. After the absorption of some pleural effusions.

*Thoracoplasty.*—Collapse operations are now frequently employed in the treatment of chronic pulmonary tuberculosis of the productive type, especially when cavitation is present. The great underlying principle of these operations should be to provide rest and relaxation to the lung. The chief indications for thoracoplasty are in unilateral disease in people between the ages of eighteen and forty-five. In selecting patients the general condition and temperament are important factors; tuberculosis elsewhere, apart from laryngeal lesions, may be a contra-indication. However good the collapse obtained, the operation is not so satisfactory as a really good artificial pneumothorax, except in patients with very chronic disease with marked distortion of the mediastinum and trachea and extreme narrowing of the intercostal spaces. Bilateral collapse operations of the apices are being employed fairly frequently.

### TYPES OF OPERATION PERFORMED.

1. *The classical Sauerbruch extra-pleural paravertebral thoracoplasty.*—For chronic fibrotic unilateral lesions with extreme lung involvement this operation provides brilliant results with low mortality rates. Such patients have an obvious resistance to their disease, and the excision of posterior segments of ribs 1 to 10 greatly benefits the natural tendency of the hemi-thorax to collapse and to shrink. The ribs are resected back to the transverse processes of the vertebræ to enable the divided anterior ends to sink backwards and downwards and also to fall in laterally in the fashion of a bucket-handle. The operation as done now is divided into two or three stages. Fig. 12 demonstrates the result of such an operation in a female patient, aged twenty-eight, who is now sputum-free and well. There is little or no obvious deformity. As good results were obtained in these favourable cases, efforts were made to close larger and larger cavities, and for this purpose it was found that larger segments of ribs required excision. For this reason the whole of the first, second and third ribs are now frequently removed.

*Apical thoracoplasty: apicolysis and Semb's operation.*—In patients with upper zone cavities it may be unnecessary to remove more than the upper ribs, the healthy basal lung being conserved. On the Continent many subclavicular cavities have been attacked by the operation of apicolysis. In this procedure the third rib is removed through a subscapular incision, the pleural dome is separated from the endothoracic fascia and overlying ribs, and the spaces so created filled with a compressing agent.

The commonest agent is paraffin wax, though fat and pectoral muscle, inserted through an anterior incision, have been used. In addition to the objection to wax as a foreign body (which may be extruded through the wound or may ulcerate through into a bronchus to be coughed up bit by bit) these compression operations are opposed to the principles of relaxation. In this country they are not considered with favour, and in the last few years have been largely replaced by two operations (*a*) Semb's extra-fascial pneumolysis and (*b*) extra-pleural artificial pneumothorax.

*The Semb operation.*—After the whole of the upper three ribs have been excised a careful dissection is made to mobilize the dome of the pleura from the stout fascial bands which pass to it from the scalene muscles. These bands, which fuse to form Sibson's fascia, pass down between the subclavian vessels and the brachial plexus. When they have been carefully divided, especially on the mediastinal aspect, the apex of the pleura with the underlying adherent lung falls to below the level of the fourth rib. The relaxation so provided has proved of the greatest value in the obliteration of large cavities, since the collapse obtained is concentric as well as lateral and antero-posterior as in the usual type of thoracoplasty. In most patients it is necessary a few weeks later to excise segments posteriorly from the fourth, fifth, sixth and seventh ribs to allow the scapula to "bed in."

*Extra-pleural artificial pneumothorax.*—This procedure appears to be of great value in the treatment of upper zone cavities and can be employed when artificial pneumothorax is indicated, but cannot be induced because of dense adhesions between the visceral and parietal pleura. Moreover, it is less mutilating and shocking than thoracoplasty and can be used when the general condition of the patient would not permit such a drastic operation. The principle of the operation is to mobilize the parietal pleura away from the endothoracic fascia at the apex, laterally, anteriorly, and on the mediastinal surface. The approach is similar to that used for apicolysis, but the extent of stripping is far greater. After all bleeding has been stopped the wound is carefully closed so as to be quite air-tight, and then an induction of an extra-pleural artificial pneumothorax is carried out to fill with air the space created by the operation (Fig. 3). Although at the beginning a little air escapes and causes subcutaneous emphysema, this soon absorbs, and an astonishingly good collapse can be obtained. At first refills are given more frequently than in intra-pleural artificial pneumothorax, but ultimately the periods can be lengthened. The operation is still in the experimental stage and it is impossible to say yet whether thoracoplasty will be required later for some of the patients. At present, however, we are pleased with the operation and hope that it will lessen the indications for the more severe operation of thoracoplasty.

### Bronchiectasis.

The most conservative physician must welcome the surgical advances in the treatment of this distressing condition. The daily misery of many of the patients is reflected in a study of their history: often children are not allowed in school because of the fœtor of their breath and the paroxysms of their cough: many of them are social pariahs from an early age: few adults with the disease can accept or obtain employment. In addition to the incapacitating symptoms and the general loss of health, their lives are constantly jeopardized by the risks of repeated pneumonia, empyema, brain abscess, and heart failure. Jex Blake's often-quoted figures show that the average expectation of life in a large series was about five years. This "medical" mortality must not be overlooked when considering that the mortality rate of lobectomy for unilateral bronchiectasis is about 14 per cent. In children under twelve years of age the mortality is far less.

*Types of bronchiectasis.*—I believe a good classification is as follows:—

1. Congenital cystic disease of the lung (not true bronchiectasis).
2. Congenital atelectatic bronchiectasis.
3. Acquired bronchiectasis.

These three types may involve one lobe, a whole lung, or be bilateral. The ideal case for lobectomy is one where the disease is confined to one lung and preferably one lobe. Success, however, is not by any means impossible even if the entire lung requires removal. In the selection of patients bronchography by means of lipiodol is essential: each side should be done separately, so that good lateral as well as antero-posterior views can be obtained and the exact state of the disease noted. Bronchoscopy and preliminary postural drainage are necessary before proceeding to lobectomy, which is now a one stage operation performed through an intercostal incision without rib resection. The essential feature of the after-treatment is the provision of underwater drainage to encourage expansion of the other lobes and to allow the cure of the empyema that often develops.

*Congenital cystic disease of the lung.*—This is by no means a rarity: the condition may involve a whole lung, one lobe, or cause solitary cyst. Symptoms such as sudden dyspnoea due to rupture of a cyst causing a spontaneous pneumothorax may come on in childhood, but more commonly cough with foul expectoration indicates an infection of the cysts. Figs. 14 and 15 show a typical example affecting the right upper lobe before and after lipiodol bronchography. Fig. 16 shows a right lower lobe removed successfully from a boy of 3½ years of age who had had a cough since infancy. Fig. 17 is a photograph of a cystic lobe removed successfully from a girl who previously had 6 to 10 ounces of foul sputum a day and who could not obtain employment: she now works as a waitress.

*Congenital atelectatic bronchiectasis.*—This is a condition seen most commonly in the left lower lobe of the lung: the history is one of persistent cough following some infantile illness. Physical signs and radiology disclose a collapsed lower lobe and lipiodol bronchography demonstrates the dilated bronchi. On a plain radiograph such lobes are triangular in outline. It is probable that such a lobe did not fully expand at or after birth and later became infected (*e.g.* during the course of measles, after broncho-pneumonia). Fig. 18 is a typical example of such a lobe: it was successfully removed from a girl of twenty who had 5 to 10 ounces of foul sputum every day for years.

*Acquired bronchiectasis.*—This is frequently a bilateral condition affecting the lower lobes and developing after pneumonia, and quite often associated with naso-pharyngeal infection. The dilatation of the bronchi is usually cylindrical. Fig. 19 represents the left lower lobe of a girl of twenty who had cough and sputum for many years, and who had had several operations on the nose and sinuses. A year after successful lobectomy she developed frontal sinusitis and died from spreading osteomyelitis of the skull. On the whole these patients are not such good surgical risks as the congenital or atelectatic types.

*Collapse operations for bronchiectasis.*—Thoracoplasty rarely succeeds in collapsing the cavities even if very wide resections have been done, and is rarely required. In one patient with right upper lobe bronchiectasis with hæmoptysis I obtained a good result after an upper thoracoplasty in which extensive segments of ribs were removed.

Phrenicectomy is a bad operation in bronchiectasis, as it fails to influence the cavities and removes power from the cough mechanism. If the nerve has been removed before lobectomy it is a serious handicap not to have a mobile diaphragm which helps the remaining lung tissue to expand. The only justification for the operation is when lipiodol bronchography shows evidence of a commencing dilatation of the bronchi in an area of unresolved pneumonia. I have been able to arrest cough and hæmoptysis in one patient in this class by phrenic avulsion.

*Lung Abscess.*—The mortality of this is distressingly high, and the time is probably coming when we shall have to abandon our rather indefinite teaching—"medical treatment for six weeks and then consider surgery." Space does not allow me to discuss treatment by early operation when the lung is in a state of early gangrene, or by lobectomy when the condition is more advanced. Many patients have apparently cured themselves by coughing up the abscesses into a bronchus (help being given by postural drainage), but a dismally high proportion of these people develop severe bronchiectasis later and often the opening into the bronchus becomes blocked by granulation tissue. Bronchoscopy is stated by some to give the best results, but over-enthusiasm will be abandoned if the after-results are carefully assessed. Drainage by surgery usually means a two-stage operation: ribs overlying the abscess are removed, and the wound packed with iodine gauze for some days to produce adhesions between the visceral and parietal pleura. The abscess is then opened widely, preferably with the cautery, and packed. The packing should be left *in situ* for some days until it separates easily, and is then removed and replaced. I have found drainage tubes to be unsatisfactory.

### THORACIC TUMOURS AND CYSTS.

This is perhaps the most interesting aspect of modern thoracic surgery. The future treatment of malignant primary growths of the lung seem to rest with total pneumonectomy: as Mr. R. C. Brock says, it is as illogical to remove a segment of breast invaded by carcinoma as to treat lung cancer by lobectomy. This field offers a wide scope for improvement in technique, but I feel sure we must persist in our efforts, for radium and deep X-ray therapy have little to offer. Innocent tumours of the bronchus are commoner than was formerly thought and can be

### PLATE V.

FIG. 16.

Cystic disease of right lower lobe removed from a child of 3½.

FIG. 17.

Cystic lobe removed by operation from a patient of 20.

FIG. 18.

Congenital atelectatic bronchiectasis: Operation specimen.

FIG. 19.

Acquired bronchiectasis: lobectomy specimen.

PLATE VI.

FIG. 20.

Radiograph of primary pleural cyst.

*(By permission of the British Journal of Surgery.)*

FIG. 21.

The cyst after excision.

*(By permission of the British Journal of Surgery.)*

recognized by bronchoscopy, which should be done in all cases of obscure hæmoptysis and cough ; they can be treated by diathermy or by a radon seed container. Fig. 13 represents the radiograph of a young woman of twenty-five who for years had suffered from a dry cough and had spent two years in a sanatorium. The appearance seen is that of a collapsed upper lobe. Bronchoscopy failed to show any obstruction, but after removal of this lobe by operation the cause of the collapse was seen to be a small innocent adenoma of the bronchus leading to the collapsed lung tissue.

*Intra-thoracic tumours that are extra-pulmonary.*—Amongst others, dermoid cysts, retro-sternal goitres, and neuro-fibromata are the cause of obstruction and compressive symptoms such as pain, dyspnoea and obstruction to the great veins, and produce cough and sputum by pressure upon the bronchi. I cannot here describe the differential diagnosis, but they offer excellent material for life-saving surgery and provide great arguments in favour of exploratory thoracotomy for doubtful shadows associated with symptoms. Fig. 20 is a slide of a man who complained of pain in the right chest, and cough ; note the circular shadow which is situated in the diaphragm and was seen on the lateral radiographs to be in the posterior mediastinum. The Casoni test for hydatid disease was negative. My preoperative diagnosis was that of a posterior mediastinal fibroma, but at operation I found and removed a primary pleural cyst (Fig. 21). I have recorded this patient and others suffering from the same condition.[2] Other conditions treated are a neuro-fibroma, a malignant tumour of the chest wall, and several patients who have been operated upon for hydatid disease of the lung. Two of these are rather rarities, as they represent the first hydatids treated by one stage lobectomy and have been recorded (d'Abreu 1937 and 1938).[3] Hydatid disease is not a rare condition in South Wales, and as many people have now crossed the Bristol Channel to live in your great city you may from time to time meet with the disease.

### REFERENCES.

[1] *The Lancet*, 11th December, 1927, p. 1,371.

[2] *British Journal of Surgery*, vol. xxv., No. 98, 1937, p. 317.

[3] *Journal International de Chirurgie*, vol. iii., No. 1, 1938, p. 1; *British Journal of Surgery*, 1937, p. 713.

### ACKNOWLEDGMENTS.

I am deeply grateful to Professor Lambert Rogers, who has given me every opportunity and encouragement; to Dr. Dan Powell, the Principal Medical Officer of the Welsh National Memorial Association, and to my senior surgical colleague in the Memorial, Mr. Price Thomas; to Dr. William Davies and Dr. D. Thomas of Sully Hospital; to Dr. Watson of Talgarth Sanatorium; Dr. Brownlee at Glan Ely; and to Drs. Gilchrist, Hiley, Trail, Carveth Johnson, Ross and Glyn Jones of the "Memorial" for their constant help and advice.

## Figures and Tables

**Fig. 1. f1:**
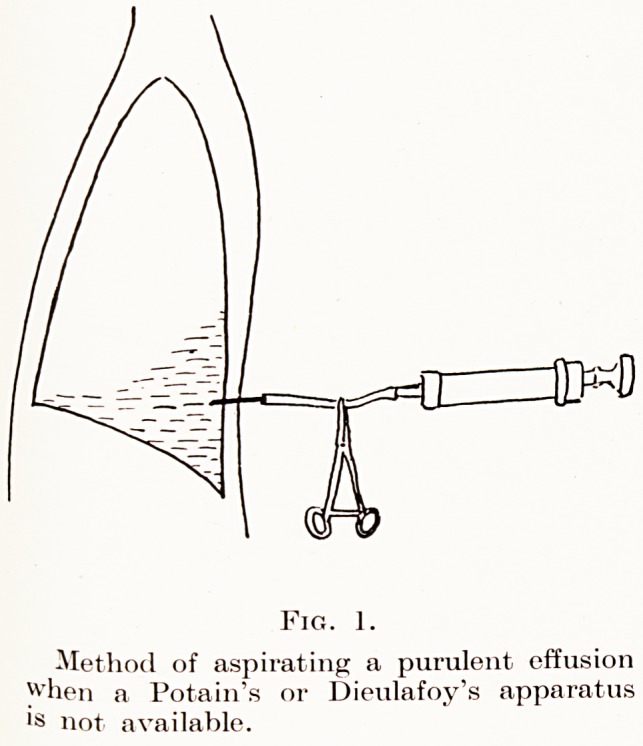


**Fig. 2. f2:**
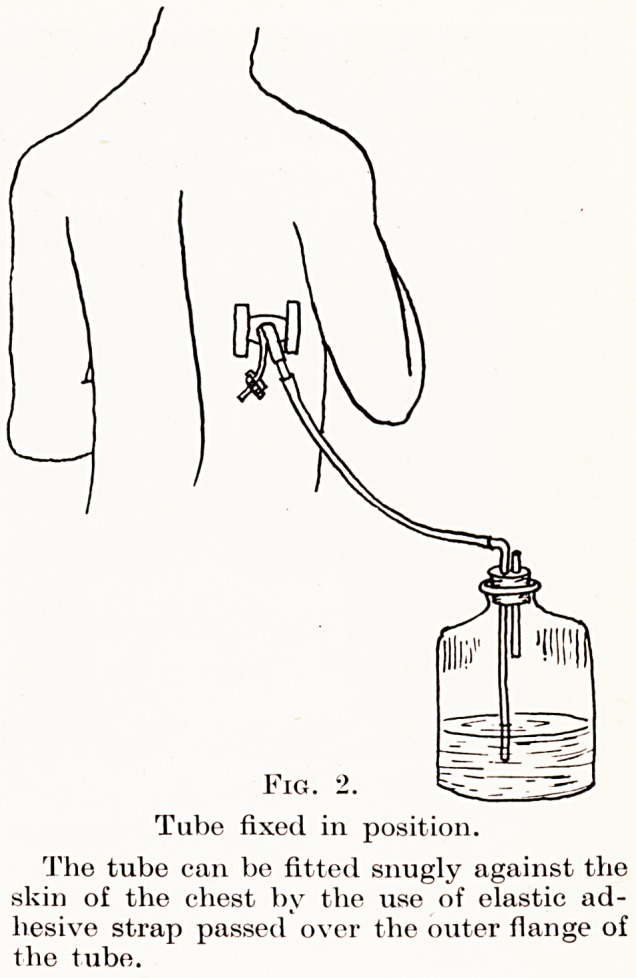


**Fig. 3. f3:**
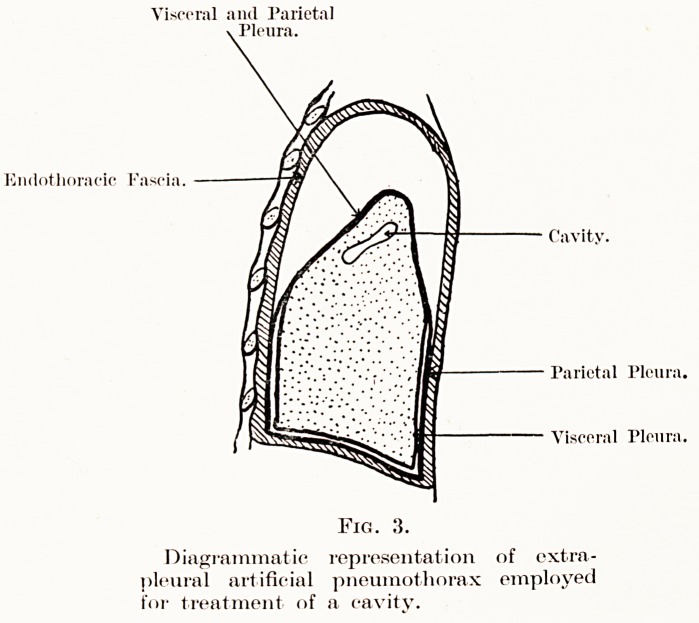


**Fig. 4. f4:**
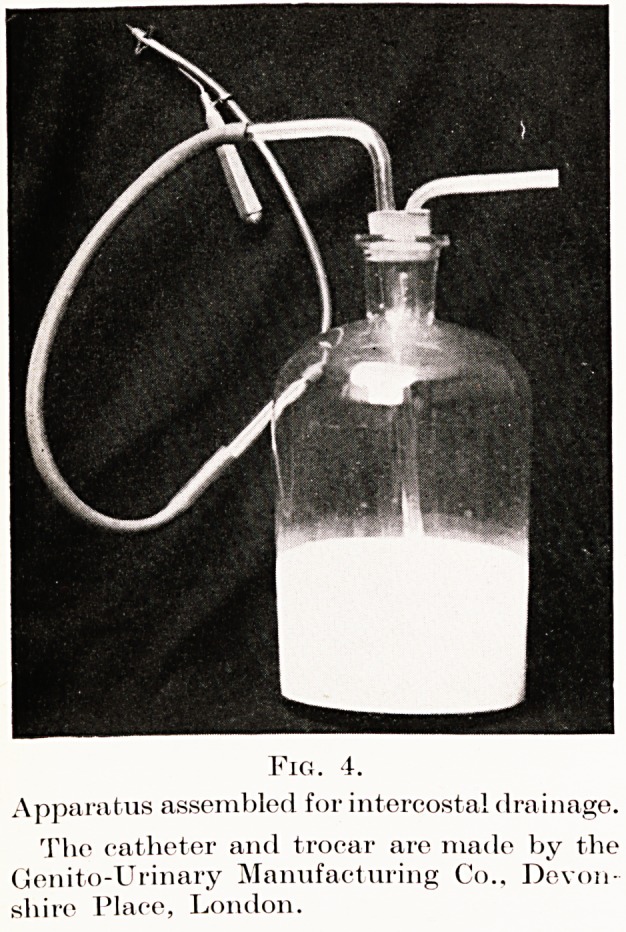


**Fig. 5. f5:**
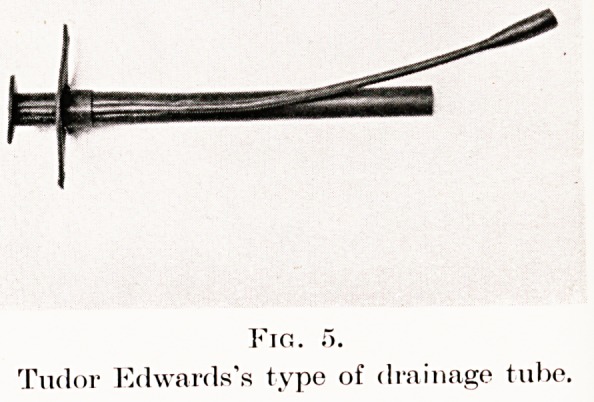


**Fig. 6. f6:**
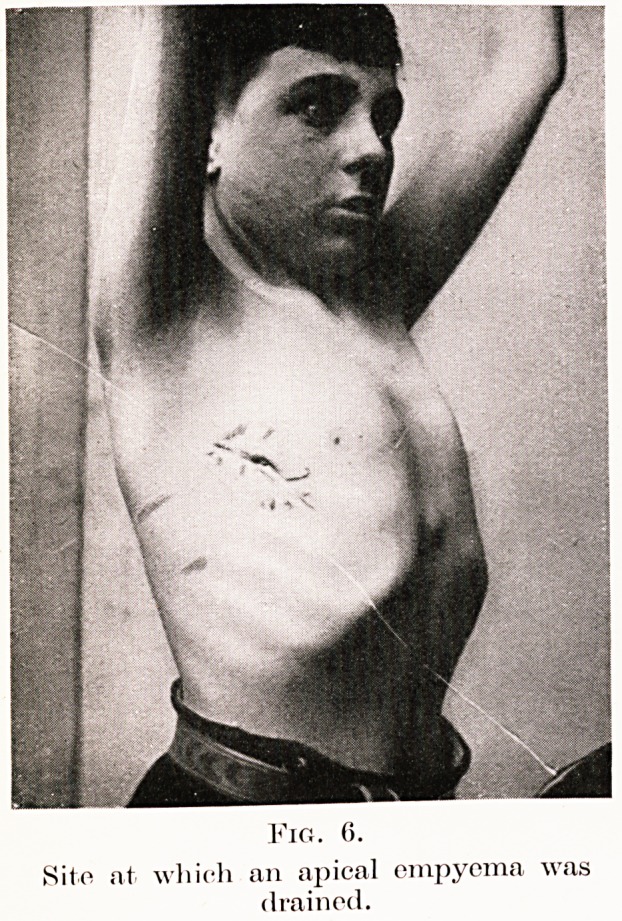


**Fig. 7. f7:**
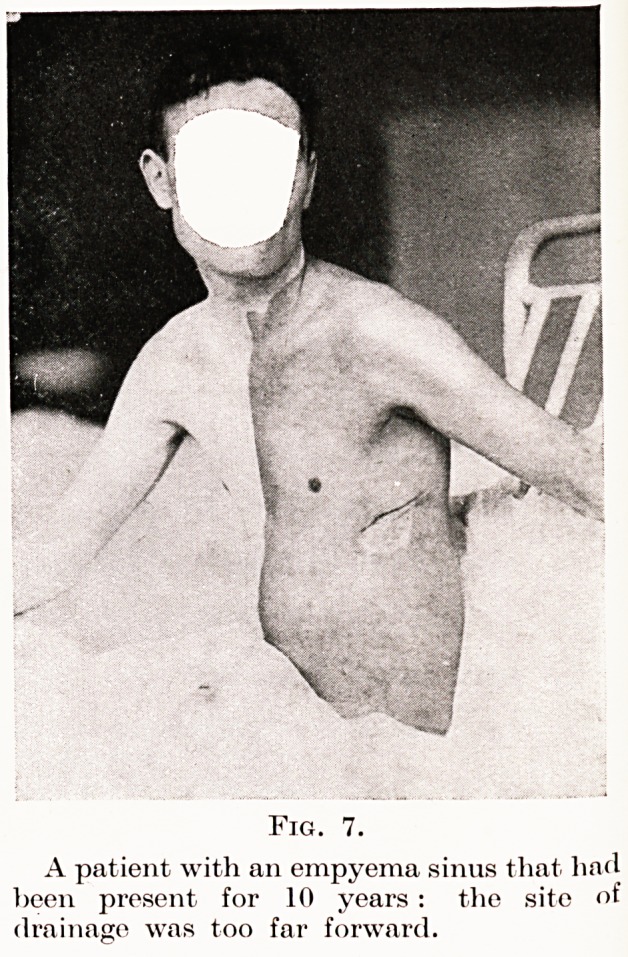


**Fig. 8. f8:**
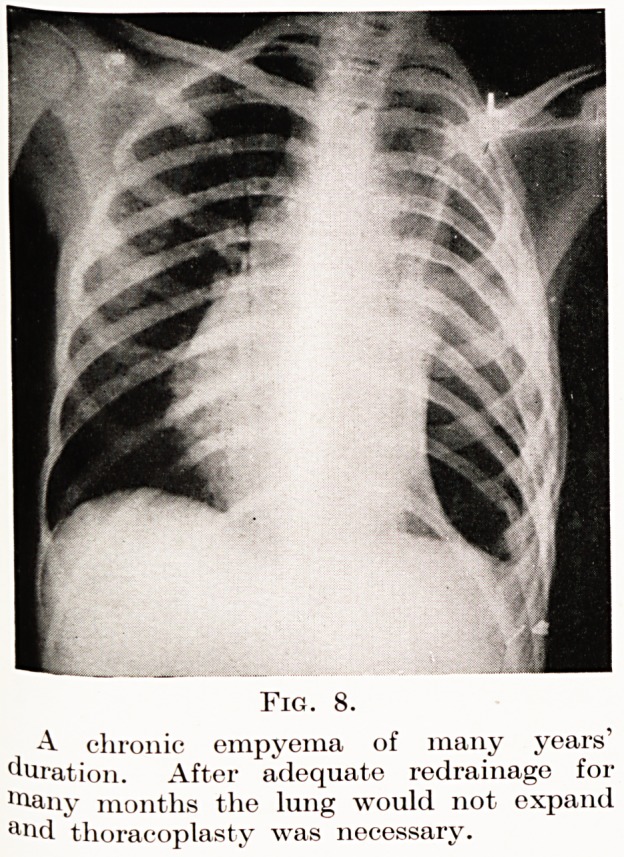


**Fig. 9. f9:**
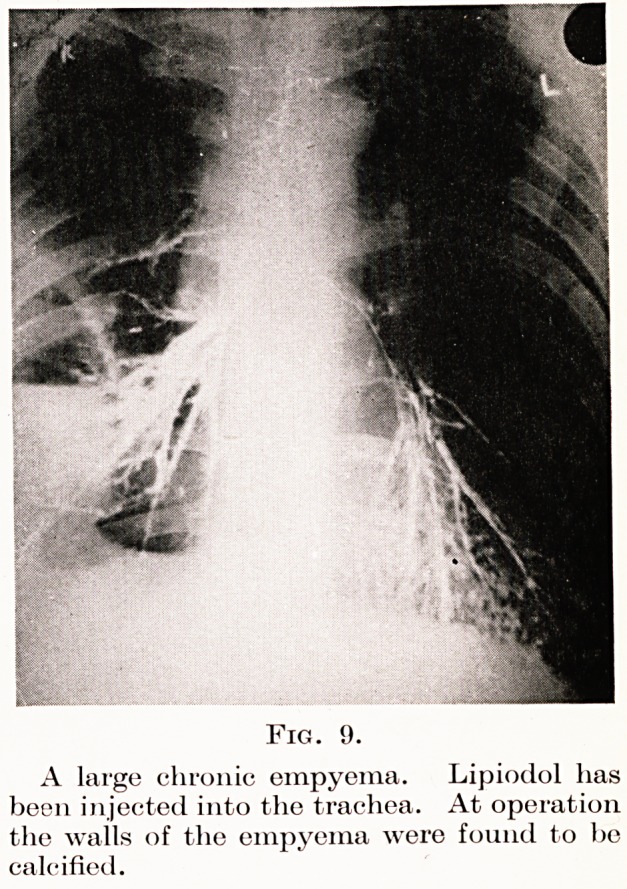


**Fig. 10. Fig. 11. f10:**
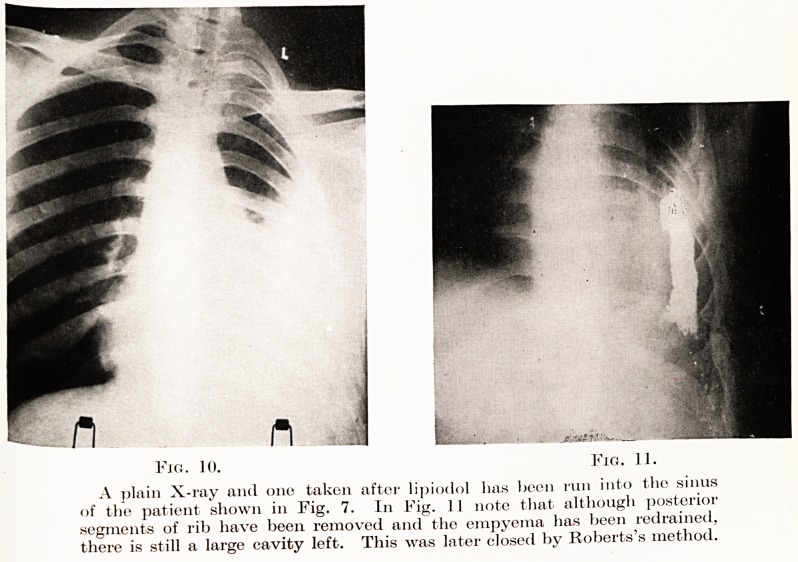


**Fig. 12. f11:**
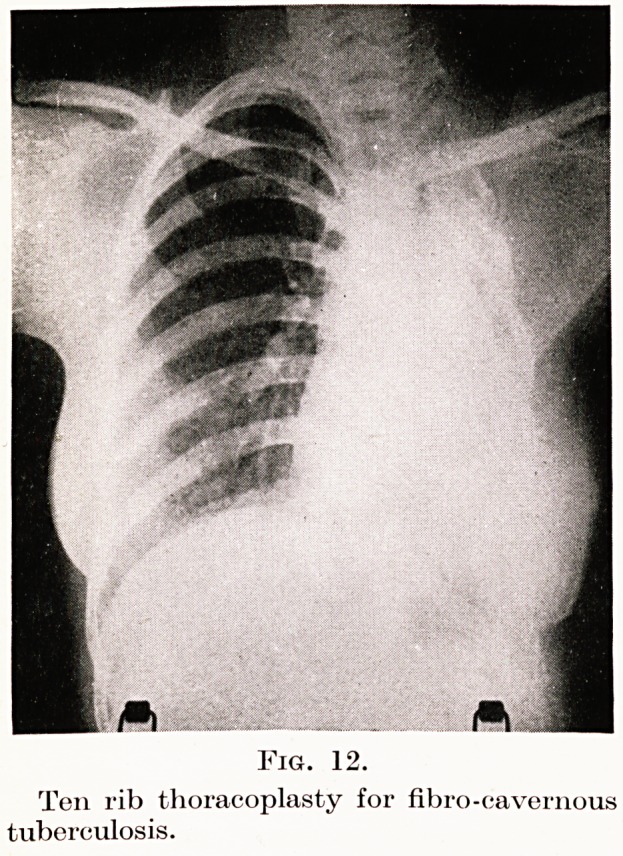


**Fig. 13. f12:**
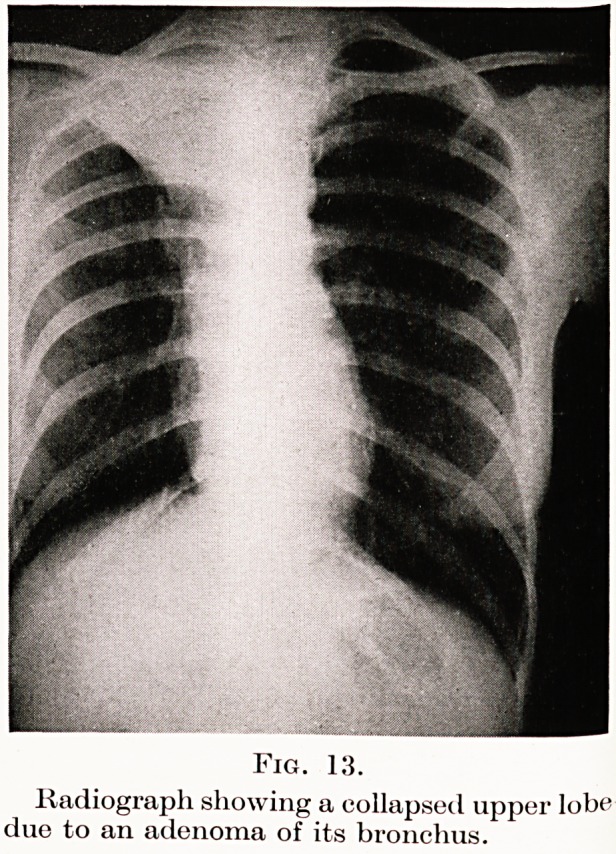


**Fig. 14. Fig. 15. f13:**
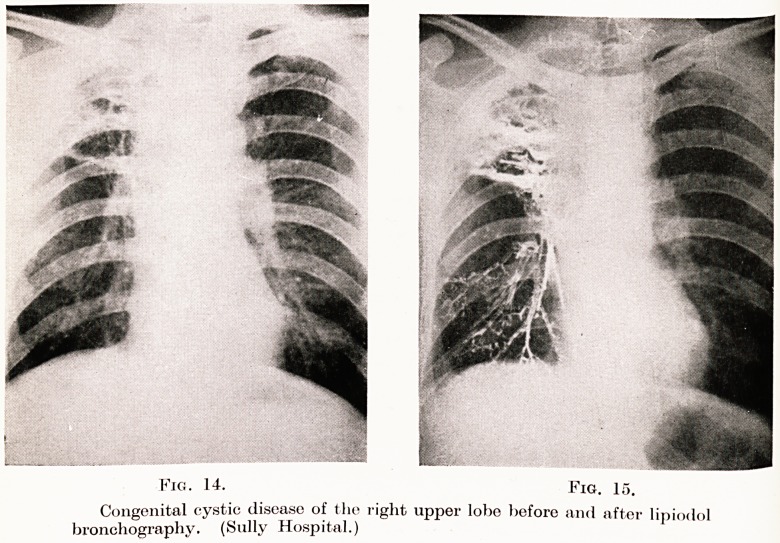


**Fig. 16. f14:**
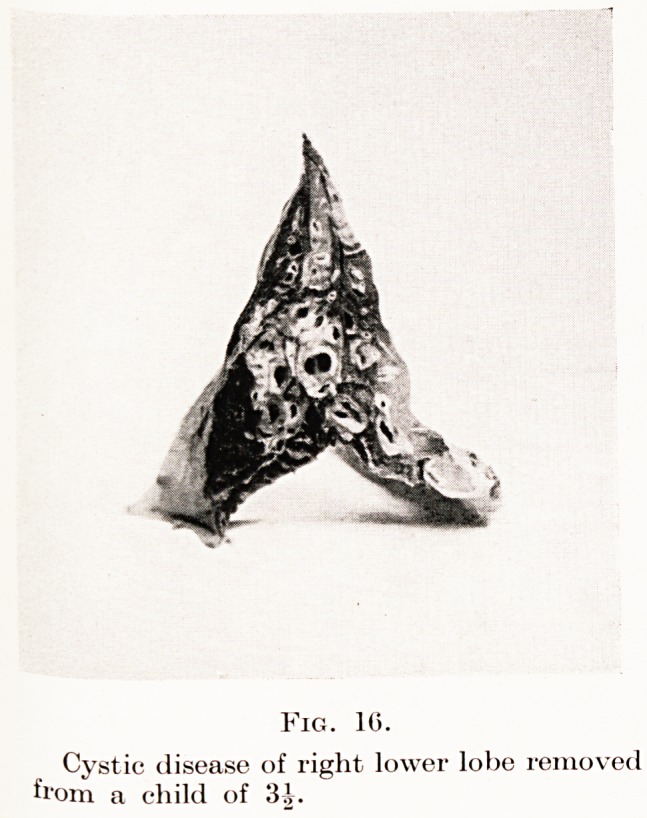


**Fig. 17. f15:**
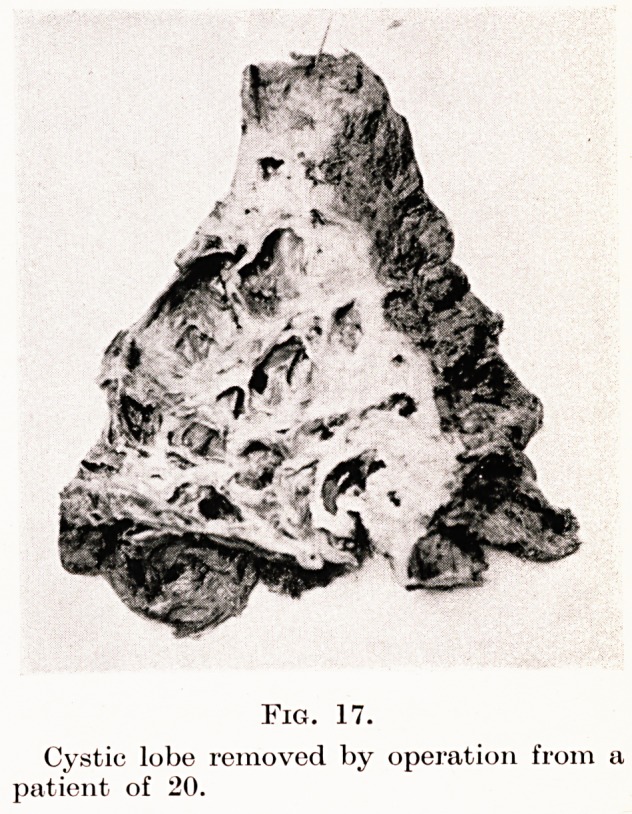


**Fig. 18. f16:**
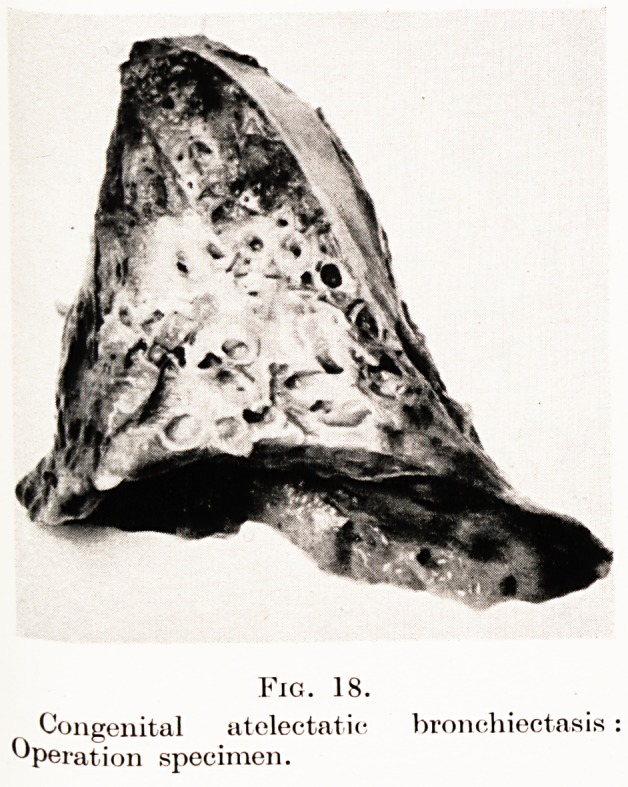


**Fig. 19. f17:**
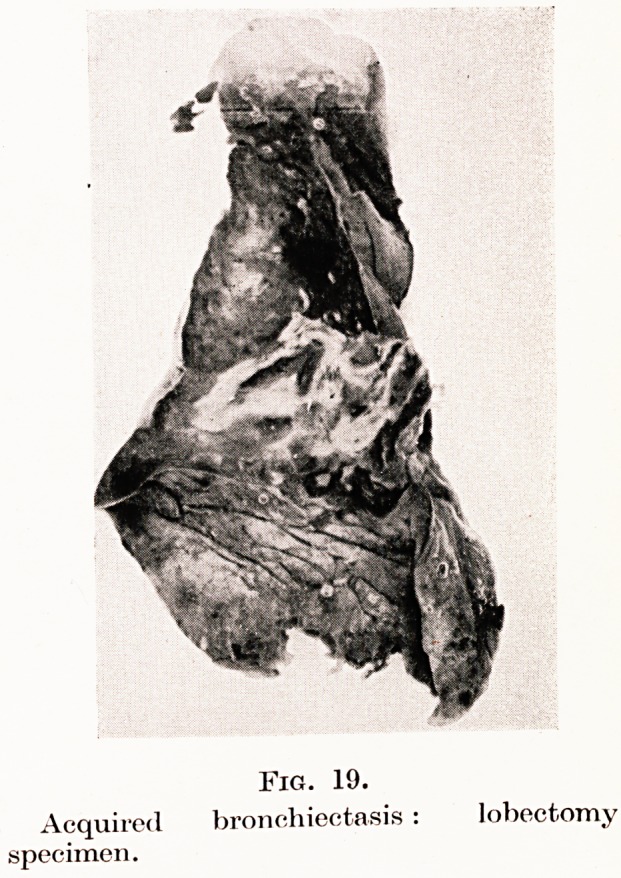


**Fig. 20. f18:**
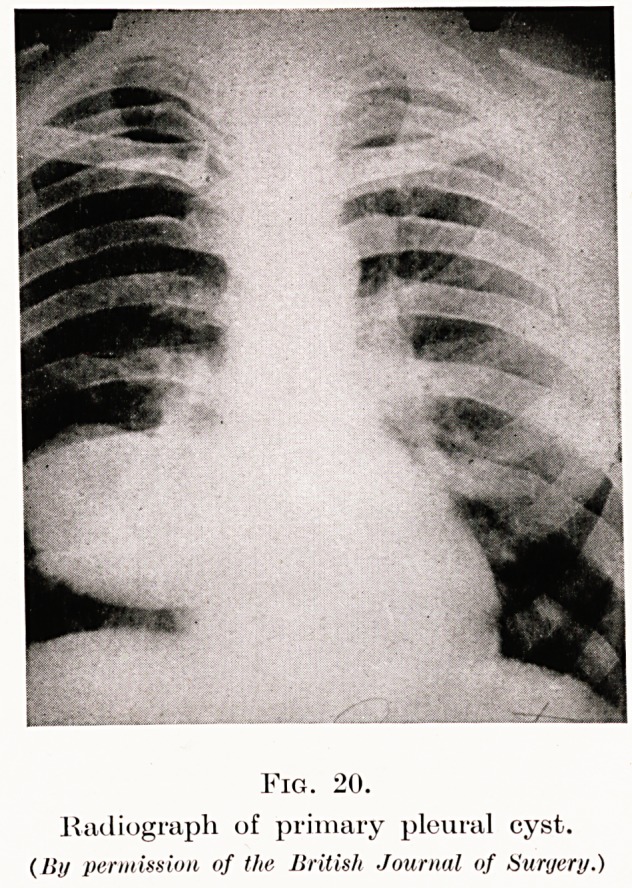


**Fig. 21. f19:**